# The chromosomal genome sequence of the Caribbean fire coral, *Millepora alcicornis* Linnaeus, 1758 (Anthoathecata: Milleporidae) and its associated microbial metagenome sequences

**DOI:** 10.12688/wellcomeopenres.27599.1

**Published:** 2026-09-17

**Authors:** Nikolaos V. Schizas, Jaaziel E. García-Hernández, Jose Victor Lopez, Nina Pruzinsky, Graeme Oatley, Elizabeth Sinclair, Eerik Aunin, Noah Gettle, Camilla Santos, Michael Paulini, Haoyu Niu, Victoria McKenna, Rebecca O’Brien, Wellcome Sanger Institute Tree of Life Management, Samples and Laboratory team, Wellcome Sanger Institute Scientific Operations: Sequencing Operations, Wellcome Sanger Institute Tree of Life Core Informatics team, EBI Aquatic Symbiosis Genomics Data Portal Team, Aquatic Symbiosis Genomics Project Leadership

**Affiliations:** 1 Department of Marine Sciences, University of Puerto Rico, Mayagüez, Puerto Rico, USA; 2 National Coral Reef Institute, Department of Biological Sciences, Nova Southeastern University, Dania Beach, Florida, USA; 3 University Corporation for Atmospheric Research (UCAR), Boulder, Colorado, USA; 4 NOAA Ocean Exploration Affiliate, Silver Spring, Maryland, USA; 5 Tree of Life Programme, Wellcome Sanger Institute, Hinxton, England, UK

**Keywords:** Millepora alcicornis, Caribbean fire coral, genome sequence, chromosomal, Anthoathecata, microbial metagenome assembly

## Abstract

We present a genome assembly from a specimen of *Millepora alcicornis* (Cnidaria; Hydrozoa; Anthoathecata; Milleporidae). The genome sequence has a total length of 3,065.30 megabases. Most of the assembly (97.85%) is scaffolded into 15 chromosomal pseudomolecules. The mitochondrial genome has also been assembled, with a length of 14.8 kilobases. Gene annotation of this assembly by Ensembl identified 18,786 protein-coding genes. From the metagenome data, we recovered four bins, of which one was a high-quality metagenome-assembled genome.

## Species taxonomy

Eukaryota; Opisthokonta; Metazoa; Eumetazoa; Cnidaria; Hydrozoa; Hydroidolina; Anthoathecata; Capitata; Milleporidae; *Millepora*; *Millepora alcicornis* Linnaeus, 1758 (NCBI:txid544493).

## Background

*Millepora alcicornis* Linnaeus, 1758, commonly known as the Caribbean fire coral ( Figure 1), is a benthic hydrocoral. It is one of the 16 recognised *Millepora* species ( WoRMS, 2026), belonging to the family Milleporidae Fleming, 1828. A key biogeographical feature of *M. alcicornis* is its status as a widespread species with an amphi-Atlantic distribution. It is mainly found in the Caribbean, but also found in Bermuda ( Sterrer, 1986), Brazil ( Amaral *et al.*, 2002, 2008), on the eastern Atlantic from the Canary Islands ( Clemente *et al.*, 2011; López *et al.*, 2015), Madeira ( Wirtz & Zilberberg, 2019), the Cape Verde Archipelago ( Boekschoten & Best, 1988) and Ascension Island ( Hoeksema *et al.*, 2017).

**Figure 1.**
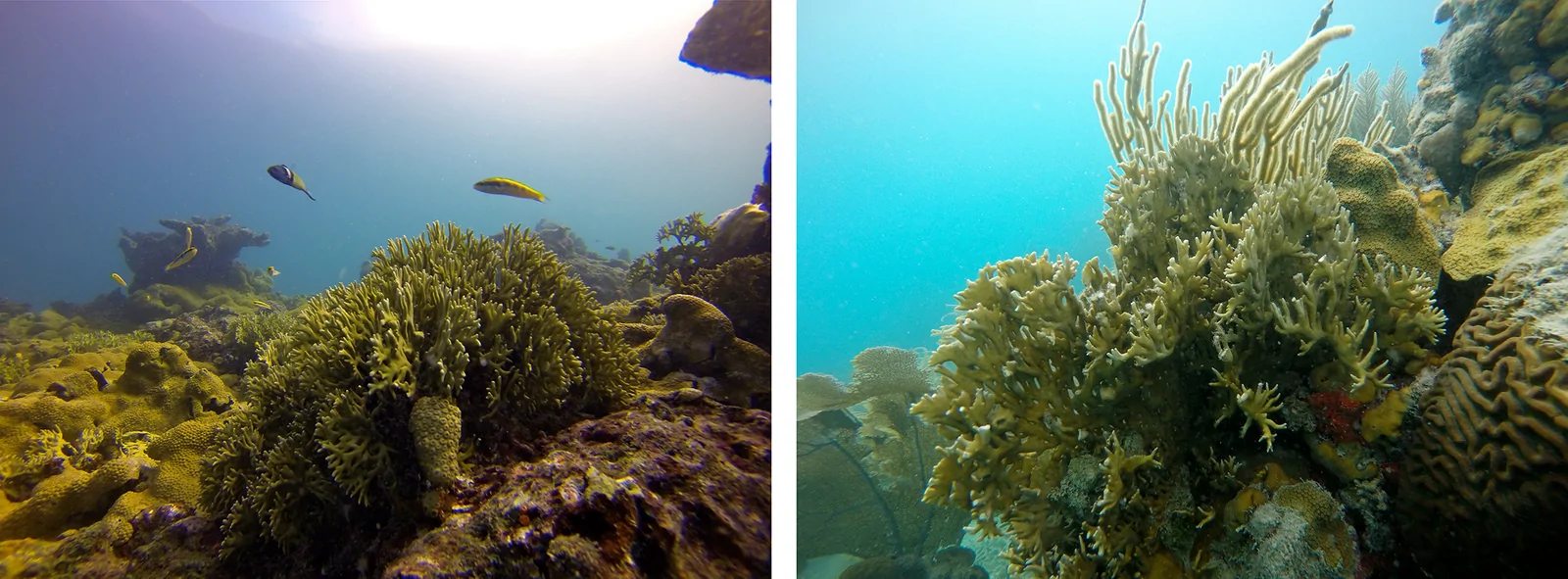
*Millepora alcicornis* colonies from La Parguera Natural Reserve, southwest Puerto Rico. All photographs by JEGH.

*Millepora alcicornis* inhabits coral reefs or even forms *Millepora* reefs, at depths ranging from 0.5 to 50 m ( de Weerdt, 1984). *M. alcicornis* colonies are commonly found in shallow, high-energy environments, where they form dense reef rims that absorb wave energy and contribute to the stabilisation and complexity of the calcium carbonate framework of Caribbean coral reefs ( Edmunds, 1999; Lewis, 2006). As coral reef degradation continues, calcium carbonate deposition by non-scleractinian organisms, such as *Millepora*, may become increasingly important for reef persistence, especially as the relative abundance of the reef-building taxa changes ( Edmunds, 2022). The species exhibits considerable phenotypic plasticity, with colony morphology adapting to local hydrodynamic conditions ( Dubé *et al.*, 2017; Edmunds, 1999). This trait underscores its adaptability across environmental gradients of light, temperature, current, and turbidity ( de Weerdt, 1984; Lewis, 1989).

*Millepora alcicornis* can be distinguished from other Caribbean milleporids by its overall colony morphology, which typically consists of upright, thin, finger-like branches that form intricate networks ( de Weerdt, 1984) ( Figure 1). This growth form creates a complex habitat for a diverse assemblage of associated fauna, including small fish, ophiuroids, barnacles, and other micro-crustaceans ( Martins Garcia *et al.*, 2008). The species demonstrates significant ecological versatility, colonising not only coral reefs but also rocky shores, seagrass beds, and the prop roots of Caribbean red mangroves ( Lewis, 2006).

*Millepora alcicornis* is capable of heterotrophy (capturing zooplankton prey with its cnidocytes) and autotrophy (through photosynthesis by its symbiotic algae) ( Lewis, 2006). *M. alcicornis* relies on both cnidocytes and secondary metabolites for its defense against predation, fouling, and overgrowth by competitors. These bioactive compounds can inhibit the growth of nearby benthic organisms and even overgrow them (scleractinian corals, gorgonians, zoanthids, and sponges) ( Lewis, 2006), allowing *M. alcicornis* to expand on the reef. The genetic pathways responsible for synthesizing these compounds are of great interest, and a de novo transcriptome assembly of *M. alcicornis* has already provided initial insights into its genetic toolkit ( Ortiz-González *et al.*, 2017). A high-quality genome assembly is the logical next step to characterise these pathways fully. Moreover, this new hydrozoan genome will facilitate further resolution of complex cnidarian phylogeny, which is composed of several lineages that do not always welcome clear resolution ( Collins, 2009; Zapata *et al.*, 2015).

*M. alcicornis* is highly vulnerable to the same threats causing the decline of scleractinian corals. It is susceptible to bleaching events, where stressful conditions – primarily elevated sea surface temperatures – cause the expulsion of its symbiotic algae (Symbiodiniaceae). The response of *M. alcicornis* to bleaching events has been well-documented, revealing significant mortality and underscoring its vulnerability to thermal stress ( Banaszak *et al.*, 2003; Vidal *et al.*, 2025). This loss of Symbiodiniaceae leads to energy deficiency, increased susceptibility to disease, and often, mortality. The increasing frequency of marine heatwaves underscores the importance of understanding the genomic underpinnings of thermal tolerance.

Furthermore, *M. alcicornis* is affected by various diseases, the prevalence of which is exacerbated by environmental stress ( Dubé *et al.*, 2017). The interaction between thermal stress, bleaching, and disease susceptibility represents a primary threat to populations across the Caribbean. Population genetic and phylogenetic studies have begun to reveal patterns of connectivity and genetic diversity ( de Souza *et al.*, 2017; Ortiz-González *et al.*, 2021; Rodríguez *et al.*, 2019; Ruiz-Ramos *et al.*, 2014). A reference genome will enable researchers to identify the specific genes and regulatory networks involved in thermal tolerance, disease resistance, and symbiosis stability, which is critical for forecasting ecological outcomes as envisioned in recent frameworks for reef futures ( Edmunds, 2022).

Population genetic studies have shown contrasting patterns of connectivity between endemic and widespread *Millepora* species in the southwestern Atlantic, with *M. alcicornis* exhibiting high levels of gene flow within the Brazilian Province, and within the greater Caribbean and Bermuda ( de Souza *et al.*, 2017). However, limited connectivity was detected between these populations and across the Atlantic ( de Souza *et al.*, 2017). Surprisingly, higher genetic relatedness was detected between western (Caribbean) and eastern Atlantic populations (Canary Islands) than between the two locations in the eastern Atlantic (Canary and Cape Verde Islands) indicating trans-oceanic connectivity ( Rodríguez *et al.*, 2019). In contrast, the Symbiodineacea of Canary Islands *M. alcicornis* were more related to those of the Cape Verde Islands, suggesting a different origin of the fire coral and its symbionts. This broad distribution, likely facilitated by long-distance dispersal of planktonic planulae during synchronised mass spawning events ( Bourmaud *et al.*, 2013; Soong & Cho, 1998), indicates a high potential for genetic exchange. The amphi-Atlantic distribution of *M.* *alcicornis* may also be attributed to transportation through ballast waters of large vessels and fouling of hulls ( Clemente *et al.*, 2011; López *et al.*, 2015). Understanding the genomic basis of local adaptation across this expansive range is crucial for predicting population-level responses to environmental change.

Hydrozoans (also hydrocorals) represent important reef builders alongside the more extensive Anthozoa. Thus, the development of a high-quality chromosomal-level genome sequence for *M. alcicornis* will build upon existing transcriptomic, population genetics, ecology and evolutionary biology research ( Leclère *et al.*, 2016; Macias-Muñoz *et al.*, 2025), unlocking unprecedented research opportunities in fire coral and hydrozoan biology. The new resource will facilitate studies into the molecular basis of calcification, the evolution of symbiosis, the mechanisms of chemical defense, and the genetic determinants of resilience.

## Methods

### Sample acquisition

Fire coral samples were collected by SCUBA within the La Parguera Natural Reserve, Cayo Mario (GPS coordinates: 17.955156, -67.05272) at a depth of 1.5 m. Tissue samples were collected under the auspices of the Puerto Rico Department of Natural and Environmental Resources, permit number R-VS-PVS15-SJ-01206-25052021. Small, 1–3 mm subsamples were cut using a sterile scalpel and preserved in RNAlater pre-labeled 2.5 ml cryogenic vials. The specimen used for PacBio sequencing had specimen ID NSU0054605, ToLID jhMilAlci5. A second specimen was used for Hi-C sequencing (specimen ID NSU0054604, ToLID jhMilAlci4). The jhMilAlci5 specimen was also used for RNA sequencing.

### Nucleic acid extraction

Protocols for high molecular weight (HMW) DNA extraction developed at the Wellcome Sanger Institute (WSI) Tree of Life Core Laboratory are available on protocols.io ( Howard *et al.*, 2025). The jhMilAlci5 sample was weighed and triaged to determine the appropriate extraction protocol. Tissue was homogenised by cryogenic disruption using the Covaris cryoPREP ^®^ Automated Dry Pulverizer. HMW DNA was extracted using the Manual MagAttract v3 protocol. The HMW DNA extraction was used for two PacBio library preparations: a standard HiFi library (TOL_ASG14776651) and a top-up ultra-low-input (ULI) library (TOL_ASG14720684). For low-input sequencing, DNA was sheared into an average fragment size of 12–20 kb following the Megaruptor ^®^3 for LI PacBio protocol. We used centrifuge-mediated fragmentation to produce DNA fragments in the 8–10 kb range, following the Covaris g-TUBE protocol for a top-up ultra-low input (ULI) library preparation. Sheared DNA was purified by automated SPRI (solid-phase reversible immobilisation). The concentration of the sheared and purified DNA from each preparation was assessed using a NanoDrop spectrophotometer and Qubit Fluorometer using the Qubit dsDNA High Sensitivity Assay kit. Fragment size distributions were evaluated by running the preparations on the FemtoPulse system. Post-shearing QC measurements were recorded separately for both PacBio library preparations. For the standard HiFi preparation (library TOL_ASG14776651), the final post-shearing DNA had a Qubit concentration of 12.2 ng/μL and an average fragment size of 14.7 kb; the 260/280 and 260/230 spectrophotometric ratios were 1.77 and 2.44, respectively. For the top-up ULI preparation (library TOL_ASG14720684), the final post-shearing DNA had a Qubit concentration of 2.6 ng/μL and an average fragment size of 9.8 kb; the 260/280 and 260/230 ratios were 1.18 and 3.39, respectively.

RNA was extracted from tissue of the jhMilAlci5 specimen in the Tree of Life Laboratory at the WSI using the RNA Extraction: Automated MagMax™ *mir*Vana protocol. The RNA concentration was assessed using a NanoDrop spectrophotometer and a Qubit Fluorometer using the Qubit RNA Broad-Range Assay kit. Analysis of the integrity of the RNA was done using the Agilent RNA 6000 Pico Kit and Eukaryotic Total RNA assay.

### PacBio HiFi library preparation and sequencing

Library preparation and sequencing were performed at the WSI Scientific Operations core. The standard HiFi and ULI libraries were sequenced in two separate runs on the Revio instrument. Libraries were prepared for PacBio - HiFi LI sequencing using the SMRTbell Prep Kit 3.0 (Pacific Biosciences, California, USA), following the manufacturer’s instructions. The kit includes reagents for end repair/A-tailing, adapter ligation, post-ligation SMRTbell bead clean-up, and nuclease treatment. Size selection and clean-up were performed using diluted AMPure PB beads (Pacific Biosciences). DNA concentration was quantified using a Qubit Fluorometer v4.0 (Thermo Fisher Scientific) and the Qubit 1X dsDNA HS assay kit. Final library fragment size was assessed with the Agilent Femto Pulse Automated Pulsed Field CE Instrument (Agilent Technologies) using the gDNA 55 kb BAC analysis kit.

For top-up sequencing, ultra-low-input (ULI) libraries were prepared using the PacBio SMRTbell ^®^ Express Template Prep Kit 2.0 and gDNA Sample Amplification Kit. Samples were normalised to 20 ng DNA. Single-strand overhang removal, DNA damage repair, and end-repair/A-tailing were performed according to the manufacturer’s instructions, followed by adapter ligation. A 0.85× pre-PCR clean-up was carried out with Promega ProNex beads.

The DNA was evenly divided into two aliquots for dual PCR (reactions A and B), both following the manufacturer’s protocol. A 0.85× post-PCR clean-up was performed with ProNex beads. DNA concentration was measured using a Qubit Fluorometer v4.0 (Thermo Fisher Scientific) with the Qubit HS Assay Kit, and fragment size was assessed on an Agilent Femto Pulse Automated Pulsed Field CE Instrument (Agilent Technologies) using the gDNA 55 kb BAC analysis kit. PCR reactions A and B were then pooled, ensuring a total mass of ≥500 ng in 47.4 μl.

The pooled sample underwent another round of DNA damage repair, end-repair/A-tailing, and hairpin adapter ligation. A 1× clean-up was performed with ProNex beads, followed by DNA quantification using the Qubit and fragment size analysis using the Agilent Femto Pulse. Size selection was performed on the Sage Sciences PippinHT system, with target fragment size determined by Femto Pulse analysis (typically 4–9 kb). Size-selected libraries were cleaned with 1.0× ProNex beads and normalised to 2 nM before sequencing.

The two libraries were sequenced in separate runs on a Revio instrument (Pacific Biosciences, California, USA). Prepared libraries with a molarity greater than 2 nM were normalised to 2 nM, and 15 μL was used for making complexes. For libraries with a molarity below 2 nM, the available 10 μL library volume was used without normalisation. Primers were annealed and polymerases were bound to generate circularised complexes, following the manufacturer’s instructions. Complexes were purified using 1.2× SMRTbell beads, diluted to the Revio loading concentration of 200–300 pM, and spiked with a Revio sequencing internal control. Each library was sequenced on a separate Revio 25M SMRT cell. SMRT Link software (Pacific Biosciences), a web-based workflow manager, was used to configure and monitor the runs and to carry out primary and secondary data analysis.

### Hi-C

***Sample preparation and crosslinking***

The Hi-C sample was prepared from 20–50 mg of frozen tissue from the jhMilAlci4 sample using the Arima-HiC v2 kit (Arima Genomics). Following the manufacturer’s instructions, tissue was fixed and DNA crosslinked using TC buffer to a final formaldehyde concentration of 2%. The tissue was homogenised using the Diagnocine Power Masher-II. Crosslinked DNA was digested with a restriction enzyme master mix, biotinylated, and ligated. Clean-up was performed with SPRISelect beads before library preparation. DNA concentration was measured with the Qubit Fluorometer (Thermo Fisher Scientific) and Qubit HS Assay Kit. The biotinylation percentage was estimated using the Arima-HiC v2 QC beads.

***Hi-C library preparation and sequencing***

Biotinylated DNA constructs were fragmented using a Covaris E220 sonicator and size-selected to 400–600 bp using SPRISelect beads. DNA was enriched with Arima-HiC v2 kit Enrichment beads. End repair, A-tailing, and adapter ligation were carried out with the NEBNext Ultra II DNA Library Prep Kit (New England Biolabs), following a modified protocol where library preparation occurs while DNA remains bound to the Enrichment beads. Library amplification was performed using KAPA HiFi HotStart mix and a custom Unique Dual Index (UDI) barcode set (Integrated DNA Technologies). Depending on sample concentration and biotinylation percentage determined at the crosslinking stage, libraries were amplified with 10–16 PCR cycles. Post-PCR clean-up was performed with SPRISelect beads. Libraries were quantified using the AccuClear Ultra High Sensitivity dsDNA Standards Assay Kit (Biotium) and a FLUOstar Omega plate reader (BMG Labtech).

Prior to sequencing, libraries were normalised to 10 ng/μL. Normalised libraries were quantified again to create equimolar and/or weighted 2.8 nM pools. Pool concentrations were checked using the Agilent 4200 TapeStation (Agilent) with High Sensitivity D500 reagents before sequencing. Sequencing was performed using paired-end 150 bp reads on the Illumina NovaSeq X.

### RNA library preparation and sequencing

Libraries were prepared using the NEBNext ^®^ Ultra II Directional RNA Library Prep Kit for Illumina (New England Biolabs), following the manufacturer’s instructions. Poly(A) mRNA was isolated from 100 ng of total RNA using oligo (dT) beads, converted to cDNA, and uniquely indexed; 14 PCR cycles were performed. Libraries were prepared with an intended average fragment size of 100–300 bp. Libraries were quantified, normalised, and pooled equimolarly to a final concentration of 2.8 nM before dilution to 150 pM for loading. Sequencing was carried out on the Illumina NovaSeq X, generating paired-end reads.

### Genome assembly

Prior to assembly of the PacBio HiFi reads, a database of *k*-mer counts ( *k* = 31) was generated from the filtered reads using FastK. GenomeScope2 ( Ranallo-Benavidez *et al.*, 2020) was used to analyse the *k*-mer frequency distributions, providing estimates of genome size, heterozygosity, and repeat content.

The HiFi reads were assembled using Hifiasm ( Cheng *et al.*, 2021) with the --primary option. The Hi-C reads ( Rao *et al.*, 2014) were mapped to the primary contigs using bwa-mem2 ( Vasimuddin *et al.*, 2019), and the contigs were scaffolded in YaHS ( Zhou *et al.*, 2023) with the --break option for handling potential misassemblies. The scaffolded assemblies were evaluated using Gfastats ( Formenti *et al.*, 2022), BUSCO ( Manni *et al.*, 2021) and MerquryFK ( Rhie *et al.*, 2020).

The mitochondrial genome was assembled using MitoHiFi ( Uliano-Silva *et al.*, 2023). The MitoHiFi reference was the mitochondrial genome of *Spirocodon saltatrix* (NC_049868.1).

### Assembly curation

The assembly was decontaminated using the Assembly Screen for Cobionts and Contaminants ( ASCC) pipeline. TreeVal was used to generate the flat files and maps for use in curation. Manual curation was conducted primarily in PretextView and HiGlass ( Kerpedjiev *et al.*, 2018). Scaffolds were visually inspected and corrected as described by Howe *et al*. (2021). Manual corrections included 105 breaks and 143 joins. This reduced the scaffold count by 18.5%, increased the scaffold N50 by 8.5%, and reduced the total assembly length by 2.2%. The curation process is described at sanger-tol/curation-resources . PretextSnapshot was used to generate a Hi-C contact map of the final assembly.

### Assembly quality assessment

The Merqury.FK tool ( Rhie *et al.*, 2020) was run in a Singularity container ( Kurtzer *et al.*, 2017) to evaluate *k*-mer completeness and assembly quality for the primary and alternate haplotypes using the *k*-mer database ( *k* = 31) computed prior to genome assembly. The analysis outputs included assembly QV scores and completeness statistics.

The genome was analysed using the BlobToolKit pipeline, a Nextflow implementation of the earlier Snakemake version ( Challis *et al.*, 2020). PacBio reads were aligned to the assembly using minimap2 ( Li, 2018) and processed with SAMtools ( Danecek *et al.*, 2021) to generate coverage tracks. The pipeline runs BUSCO ( Manni *et al.*, 2021) using lineages identified from the NCBI Taxonomy ( Schoch *et al.*, 2020). For the three basal BUSCO lineages, eukaryota, bacteria and archaea, BUSCO genes are aligned to the UniProt Reference Proteomes database ( Bateman *et al.*, 2023) using DIAMOND BLASTp ( Buchfink *et al.*, 2021). The genome assembly is divided into chunks based on the density of BUSCO genes from the closest taxonomic lineage, and each chunk is aligned to the UniProt Reference Proteomes database using DIAMOND BLASTx. Sequences without DIAMOND BLASTx hits are extracted using seqtk and aligned to the NT database using BLASTn ( Altschul *et al.*, 1990). The outputs are consolidated into a BlobDir for visualisation in BlobToolKit. The BlobToolKit pipeline was developed using nf-core tooling ( Ewels *et al.*, 2020) and MultiQC ( Ewels *et al.*, 2016), with containerisation through Docker ( Merkel, 2014) and Singularity ( Kurtzer *et al.*, 2017).

### Metagenome assembly

The metagenome assembly was generated using MetaMDBG ( Benoit *et al.*, 2024) and binned using maxbin2. The resulting bin sets of each binning algorithm were optimised and refined using DAS Tool ( Sieber *et al.*, 2018). PROKKA ( Seemann, 2014) was used to identify tRNAs and rRNAs in each bin, CheckM ( Parks *et al.*, 2015) (checkM_DB release 2015-01-16) was used to assess bin completeness/contamination, and GTDB-Tk ( Chaumeil *et al.*, 2022) (GTDB release 214) was used to taxonomically classify bins. Taxonomic replicate bins were identified using dRep ( Olm *et al.*, 2017) with default settings (95% ANI threshold). All bins were assessed for quality and categorised as metagenome-assembled genomes (MAGs) if they met the following criteria: contamination ≤ 5%, presence of 5S, 16S, and 23S rRNA genes, at least 18 unique tRNAs, and either ≥ 90% completeness or ≥ 50% completeness with fully circularised chromosomes ( Bowers *et al.*, 2017). Bins that did not meet these thresholds, or were identified as taxonomic replicates of MAGs, were retained as ‘binned metagenomes’ provided they had ≥ 50% completeness and ≤ 10% contamination. A taxonomic tree of the bins was constructed from NCBI classifications using ete3 ( Huerta-Cepas *et al.*, 2016) and visualised with matplotlib.

## Genome sequence report

### Sequence data

PacBio sequencing of the *Millepora alcicornis* specimen generated 160.08 Gb (gigabases) from 14.99 million reads, which were used to assemble the genome. GenomeScope2.0 analysis using a *p* = 2 model gave a *k*-mer-based genome size estimate of 2,977.48 Mb, with a heterozygosity of 1.04% and repeat content of 31.87% ( Figure 2). These estimates guided expectations for the assembly. Based on the estimated genome size, the sequencing data provided approximately 50× coverage. Hi-C sequencing produced 117.01 Gb from 387.47 million reads, which were used to scaffold the assembly. RNA sequencing data were also generated and are available in public sequence repositories. Table 1 summarises the specimen and sequencing details.

**Figure 2.**
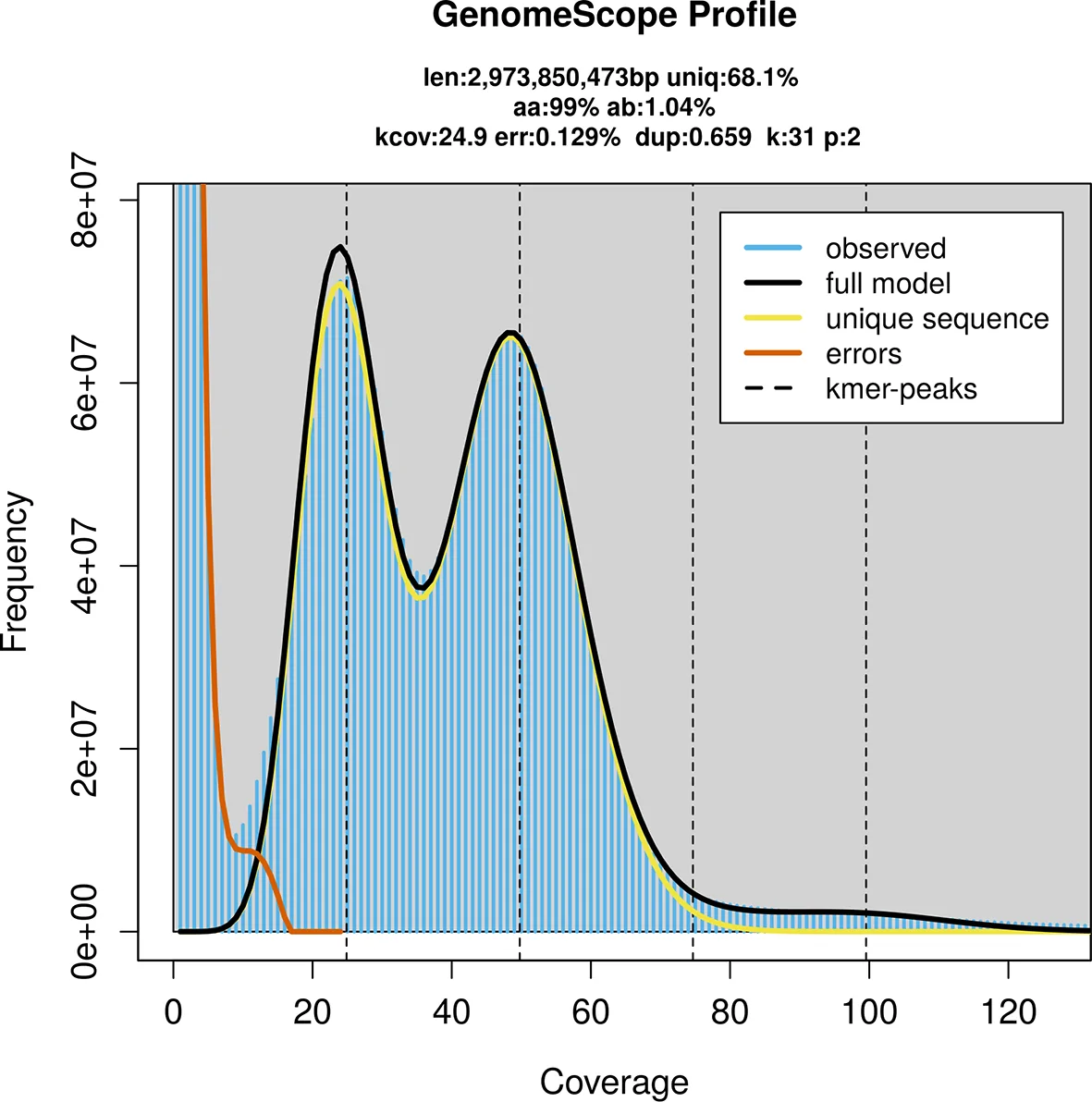
Frequency distribution of *k*-mers generated using GenomeScope2. The plot shows observed and modelled *k*-mer spectra, providing estimates of genome size, heterozygosity, and repeat content based on unassembled sequencing reads.

**Table 1.** Specimen and sequencing data for *Millepora alcicornis* (BioProject PRJEB83134).

Platform	PacBio HiFi	Hi-C	RNA-seq
**ToLID**	jhMilAlci5	jhMilAlci4	jhMilAlci5
**Specimen ID**	NSU0054605	NSU0054604	NSU0054605
**BioSample (source individual)**	SAMEA12927248	SAMEA12927247	SAMEA12927248
**BioSample (tissue)**	SAMEA12927296	SAMEA12927295	SAMEA12927296
**Tissue**	other somatic animal tissue	other somatic animal tissue	other somatic animal tissue
**Instrument**	Revio	Illumina NovaSeq X	Illumina NovaSeq X
**Run accessions**	ERR14048070; ERR14048069	ERR14056183	ERR14056182
**Read count total**	14.99 million reads	387.47 million read pairs	48.15 million read pairs
**Base count total**	160.08 Gb	117.01 Gb	14.54 Gb

### Assembly statistics

The primary haplotype was assembled, and scaffolds corresponding to an alternate haplotype were also deposited in INSDC databases. The final assembly has a total length of 3,065.30 Mb in 444 scaffolds, with 562 gaps, and a scaffold N50 of 198.42 Mb ( Table 2).

**Table 2.** Genome assembly data for *Millepora alcicornis.*

Genome assembly	Primary assembly
**Assembly name**	jhMilAlci5.1
**Assembly accession**	GCA_965112645.1
**Alternate haplotype accession**	GCA_965112675.1
**Assembly level**	chromosome
**Span (Mb)**	3,065.30
**Number of chromosomes**	15
**Number of contigs**	1,006
**Contig N50 (Mb)**	10.03
**Number of scaffolds**	444
**Scaffold N50 (Mb)**	198.42
**Organelles**	Mitochondrial genome: 14.80 kb

Most of the assembly sequence (97.85%) was assigned to 15 chromosome-level scaffolds. These chromosome-level scaffolds, confirmed by Hi-C data, are named according to size ( Figure 3; Table 3). The exact order and orientation of the contigs are unknown in the following regions: chromosome 1 (137,500–139,500 Kbp), chromosome 3 (92,700–98,200 Kbp), chromosome 4 (81,000–85,000 Kbp), chromosome 5 (86,500–94,100 Kbp), chromosome 7 (108,600–120,000 Kbp), chromosome 10 (63,800–66,500 Kbp), chromosome 11 (82,300–86,900 Kbp), and chromosome 13 (86,300–91,000 Kbp).

**Figure 3.**
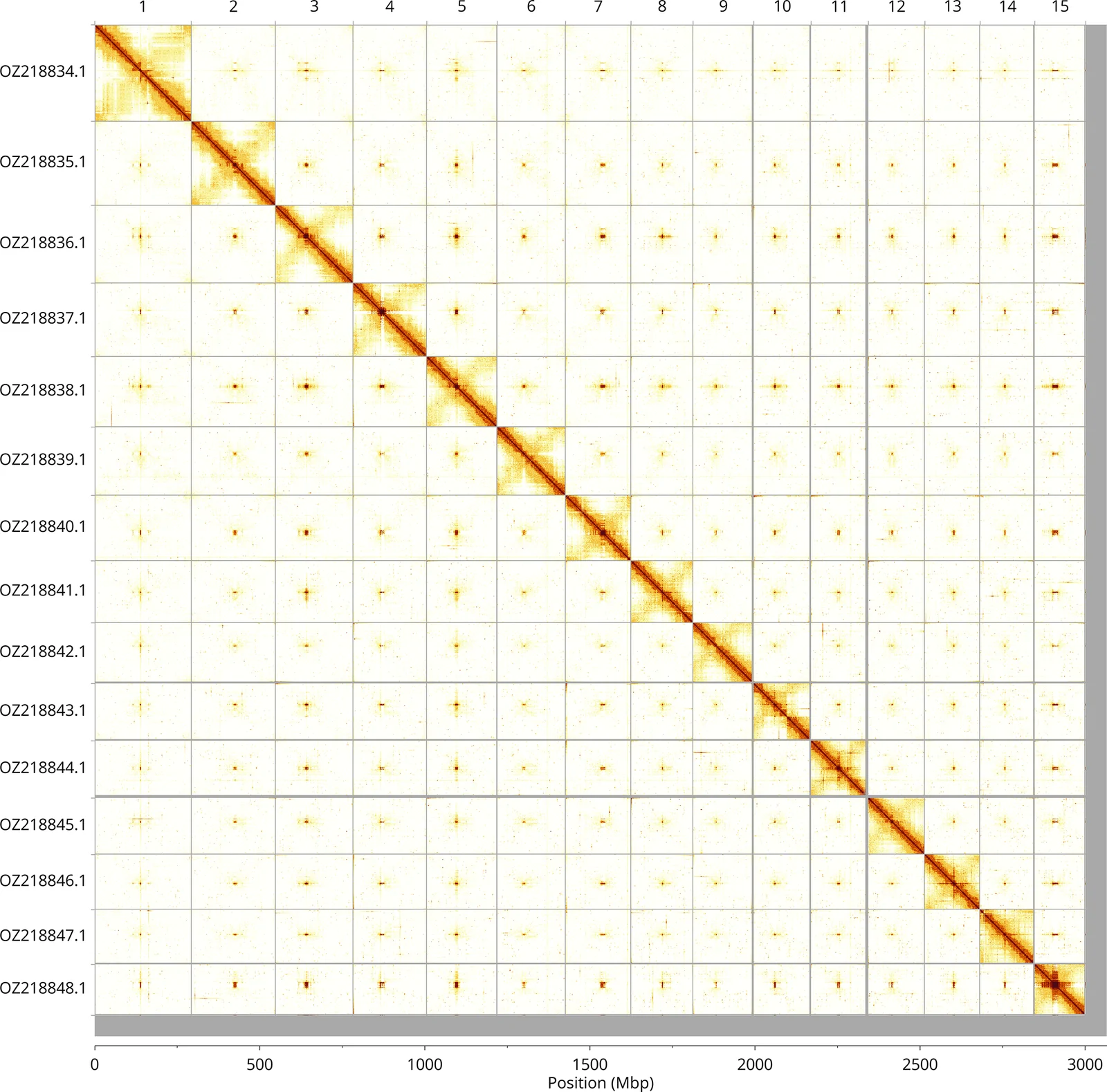
Hi-C contact map of the *Millepora alcicornis* genome assembly. Assembled chromosomes are shown in order of size and labelled along the axes, with a megabase scale shown below. The plot was generated using PretextSnapshot.

**Table 3.** Chromosomal pseudomolecules in the primary genome assembly of *Millepora alcicornis* jhMilAlci5.

INSDC accession	Molecule	Length (Mb)	GC%
OZ218834.1	1	292.32	31.50
OZ218835.1	2	254.97	31.50
OZ218836.1	3	235.17	32
OZ218837.1	4	222.70	32
OZ218838.1	5	213.17	32
OZ218839.1	6	207	32
OZ218840.1	7	198.42	32
OZ218841.1	8	187.68	31.50
OZ218842.1	9	183.21	31.50
OZ218843.1	10	173.59	32
OZ218844.1	11	173.43	31.50
OZ218845.1	12	171.65	31.50
OZ218846.1	13	167.10	31.50
OZ218847.1	14	165.23	31.50
OZ218848.1	15	153.83	32

The mitochondrial genome was also assembled (length 14.8 kb, OZ218849.1). This sequence is included as a contig in the multifasta file of the genome submission and as a standalone record.

### Assembly quality metrics

The combined primary and alternate assemblies achieve an estimated QV of 61.1. The *k*-mer completeness is 80.30% for the primary assembly, 79.82% for the alternate haplotype, and 99.03% for the combined assemblies ( Figure 4).

**Figure 4.**
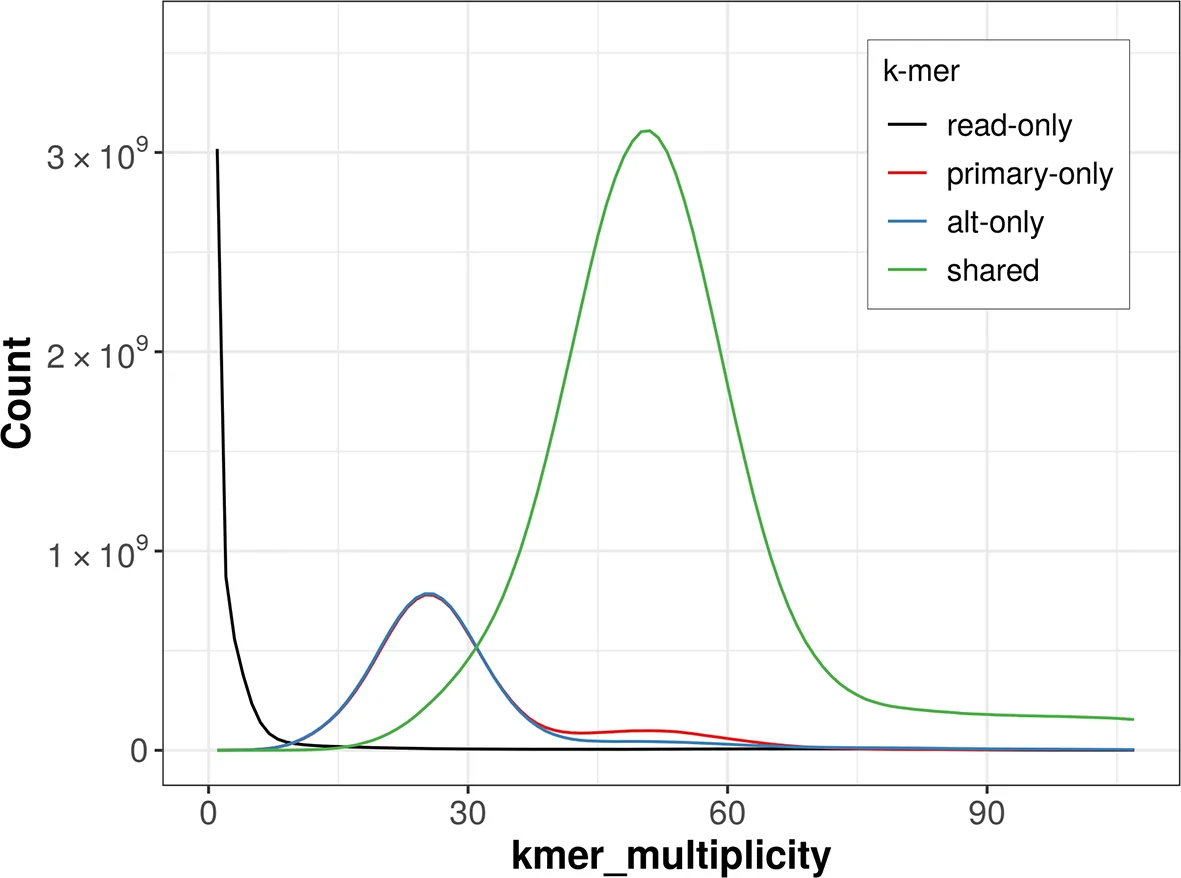
Evaluation of *k*-mer completeness using MerquryFK. This plot illustrates the recovery of *k*-mers from the original read data in the final assemblies. The horizontal axis represents *k*-mer multiplicity, and the vertical axis shows the number of *k*-mers. The black curve represents *k*-mers that appear in the reads but are not assembled. The green curve corresponds to *k*-mers shared by both haplotypes, and the red and blue curves show *k*-mers found only in one of the haplotypes.

BUSCO v.5.7.1 analysis using the metazoa_odb10 reference set ( *n* = 954) identified 89.0% of the expected gene set (single = 87.0%, duplicated = 2.0%). The snail plot in Figure 5 summarises the scaffold length distribution and other assembly statistics for the primary assembly. The blob plot in Figure 6 shows the distribution of scaffolds by GC proportion and coverage.

**Figure 5.**
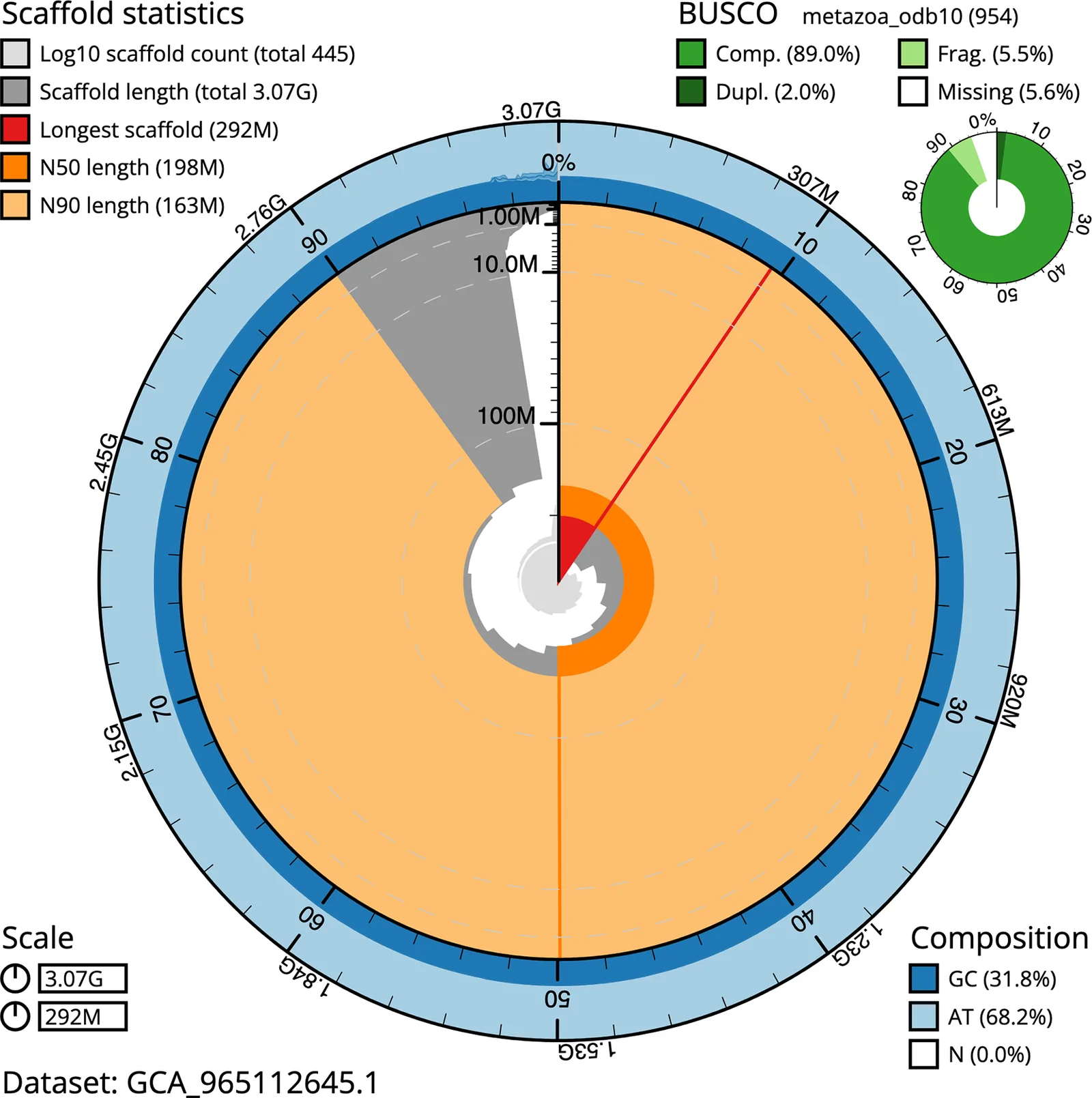
Assembly metrics for jhMilAlci5.1. The BlobToolKit snail plot provides an overview of assembly metrics and BUSCO gene completeness. The circumference represents the length of the whole genome sequence, and the main plot is divided into 1 000 bins around the circumference. The outermost blue tracks display the distribution of GC, AT, and N percentages across the bins. Scaffolds are arranged clockwise from longest to shortest and are depicted in dark grey. The longest scaffold is indicated by the red arc, and the deeper orange and pale orange arcs represent the N50 and N90 lengths. A light grey spiral at the centre shows the cumulative scaffold count on a logarithmic scale. A summary of complete, fragmented, duplicated, and missing BUSCO genes in the metazoa_odb10 set is presented at the top right. An interactive version of this figure can be accessed on the BlobToolKit viewer.

**Figure 6.**
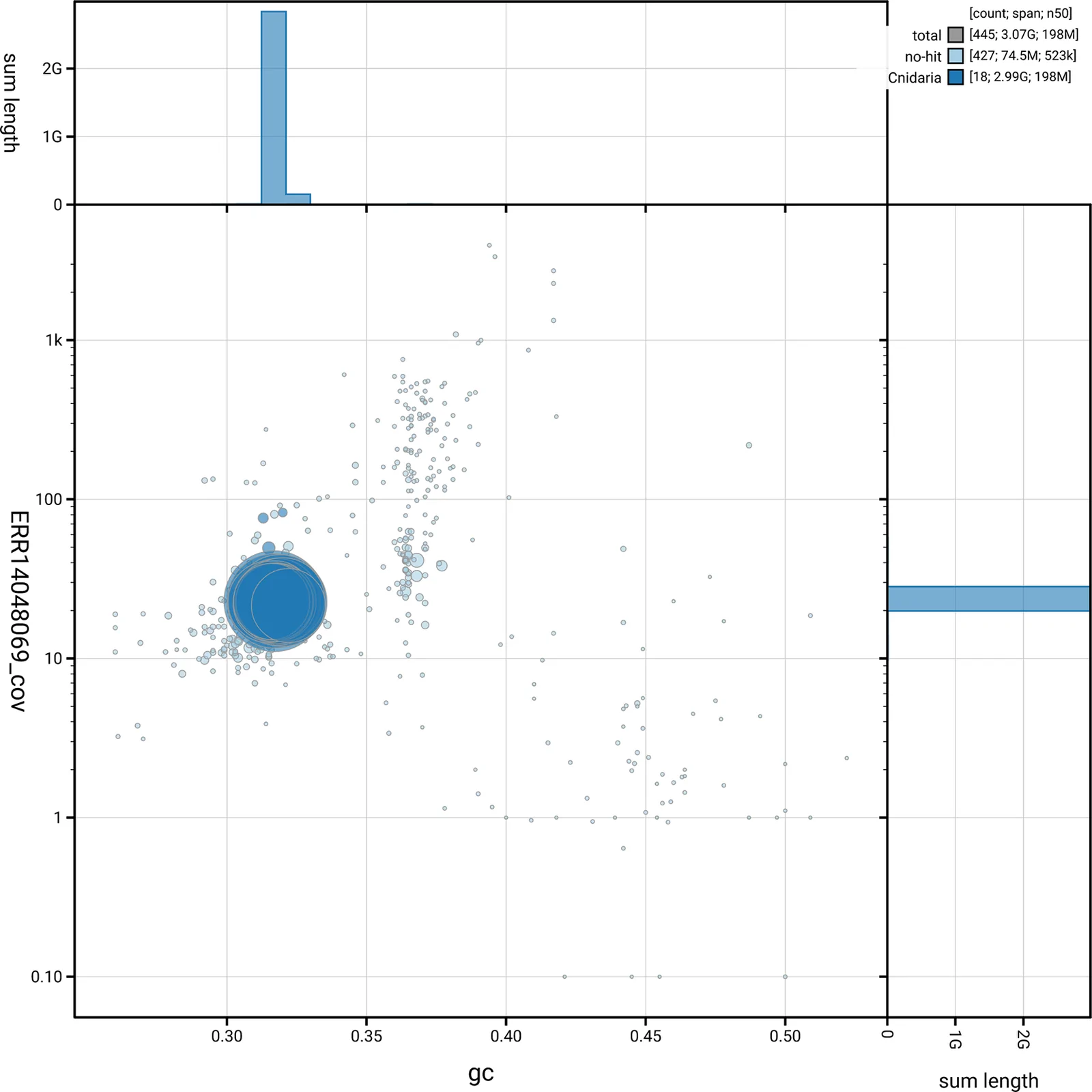
BlobToolKit blob plot for jhMilAlci5.1. The plot shows base coverage (vertical axis) and GC content (horizontal axis). The circles represent scaffolds, with the size proportional to scaffold length and the colour representing phylum membership. The histograms along the axes display the total length of sequences distributed across different levels of coverage and GC content. An interactive version of this figure is available on the BlobToolKit viewer.

Table 4 lists the assembly metric benchmarks adapted from Rhie *et al.* (2021) and the Earth BioGenome Project (EBP) Report on Assembly Standards January 2026. The EBP metric, calculated for primary assembly, is **7.C.Q61**, meeting the recommended 6.C.Q40 reference standard.

**Table 4.** Earth Biogenome Project summary metrics for the *Millepora alcicornis* assembly.

Measure	Value	Benchmark
EBP summary (primary)	7.C.Q61	6.C.Q40
Contig N50 length	10.03 Mb	≥ 1 Mb
Scaffold N50 length	198.42 Mb	= chromosome N50
Consensus quality (QV)	Primary: 61.5; alternate: 60.7; combined: 61.1	≥ 40
*k*-mer completeness	Primary: 80.30%; alternate: 79.82%; combined: 99.03%	> 90%
BUSCO	C:89.0% [S:87.0%, D:2.0%], F:5.5%, M:5.6%, n:954	S > 90%; D < 5%
Percentage of assembly assigned to chromosomes	97.85%	≥ 90%

*Note*: The EBP summary uses log10(Contig N50); chromosome-level (C) or log10(Scaffold N50); Q (Merqury QV). BUSCO: C=complete; S=single-copy; D=duplicated; F=fragmented; M=missing; n=orthologues

### Genome annotation report

The *Millepora alcicornis* genome assembly (GCA_965112645.1) was annotated by Ensembl at the European Bioinformatics Institute (EBI). This annotation includes 42,318 transcribed mRNAs from 18,786 protein-coding and 17,385 non-coding genes. The average transcript length is 18,500.71 bp, with an average of 1.17 coding transcripts per gene and 3.76 exons per transcript. Further details of this annotation are available from the Ensembl annotation page.

### Metagenome report

We recovered four bins from the metagenome assembly ( Table 5), of which one met the criteria for a MAG. The recovered bins represented one bacterial phyla, with genome sizes ranging from 2.11 to 4.40 Mbp (mean: 3.16 ± 0.94 Mbp). Mean completeness was 60.1% (± 22.2%) with 4.5% (± 2.5%) contamination.

**Table 5.** Quality metrics and taxonomic assignments of the binned metagenomes.

NCBI taxon	GTDB taxonomy	Quality	Size (bp)	Contigs	Circular	Mean coverage	Completeness (%)	Contamination (%)
Gammaproteobacteria bacterium	Gammaproteobacteria	MEDIUM	2,403,596	116	No	3.32	58.27	8.29
Congregibacter sp.	Gammaproteobacteria	MEDIUM	4,402,211	120	No	3.05	57.99	4.81
Gammaproteobacteria bacterium	Gammaproteobacteria	LOW	2,114,542	119	No	2.81	30.88	3.04
Gammaproteobacteria bacterium	Gammaproteobacteria	HIGH	3,736,913	1	Yes	9.68	93.37	1.76

**Table 6.** Software versions and sources used for *Millepora alcicornis.*

Software	Version	Source
BLAST	2.14.0	https://ftp.ncbi.nlm.nih.gov/blast/executables/blast+/
blobtk	0.5.1	https://github.com/genomehubs/blobtk
BlobToolKit	4.4.5	https://github.com/genomehubs/blobtoolkit
BUSCO	5.7.1	https://gitlab.com/ezlab/busco
bwa-mem2	2.2.1	https://github.com/bwa-mem2/bwa-mem2
CheckM	2015-01-16	https://ecogenomics.github.io/CheckM/
DAS Tool	1.1.2	https://github.com/cmks/DAS_Tool
DIAMOND	2.1.8	https://github.com/bbuchfink/diamond
dRep	3.4.0	https://github.com/MrOlm/drep
ete3	3.1.3	https://etetoolkit.org/
fasta_windows	0.2.4	https://github.com/tolkit/fasta_windows
FastK	1.1	https://github.com/thegenemyers/FASTK
GenomeScope2.0	2.0.1	https://github.com/tbenavi1/genomescope2.0
Gfastats	1.3.6	https://github.com/vgl-hub/gfastats
GTDB-Tk	1.2.1	https://github.com/Ecogenomics/GTDBTk
Hifiasm	0.19.8-r603	https://github.com/chhylp123/hifiasm
HiGlass	1.13.4	https://github.com/higlass/higlass
matplotlib	3.10.3	https://matplotlib.org/
MaxBin	2.2.7	https://sourceforge.net/projects/maxbin/
MerquryFK	1.1.0-c1	https://github.com/thegenemyers/MERQURY.FK
MetaMDBG	Pre-release	https://github.com/GaetanBenoitDev/metaMDBG
Minimap2	2.24-r1122; 2.28-r1209	https://github.com/lh3/minimap2
MitoHiFi	3.2.2	https://github.com/marcelauliano/MitoHiFi
MultiQC	1.14; 1.17 and 1.18	https://github.com/MultiQC/MultiQC
Nextflow	23.10.0; 24.10.4	https://github.com/nextflow-io/nextflow
PretextSnapshot	0.0.6	https://github.com/sanger-tol/PretextSnapshot
PretextView	1.0.3	https://github.com/sanger-tol/PretextView
Prokka	1.14.5	https://github.com/tseemann/prokka
samtools	1.17; 1.18; 1.2; 1.21; 1.6	https://github.com/samtools/samtools
sanger-tol/ascc	0.1.0	https://github.com/sanger-tol/ascc
sanger-tol/blobtoolkit	v0.7.1	https://github.com/sanger-tol/blobtoolkit
sanger-tol/curationpretext	1.4.2	https://github.com/sanger-tol/curationpretext
Seqtk	1.4-r122	https://github.com/lh3/seqtk
Singularity	3.9.0	https://github.com/sylabs/singularity
TreeVal	1.1.1	https://github.com/sanger-tol/treeval
YaHS	1.2a.2	https://github.com/c-zhou/yahs

## Author information

Contributors are listed at the following links:

• Members of the Wellcome Sanger Institute Tree of Life Management, Samples and Laboratory team
• Members of Wellcome Sanger Institute Scientific Operations – Sequencing Operations
• Members of the Wellcome Sanger Institute Tree of Life Core Informatics team
• Members of the EBI Aquatic Symbiosis Genomics Data Portal Team
• The Aquatic Symbiosis Genomics Project leadership

## Wellcome sanger institute – legal and governance

The materials that have contributed to this genome note have been supplied by a Tree of Life collaborator. The Wellcome Sanger Institute employs a process whereby due diligence is carried out proportionate to the nature of the materials themselves, and the circumstances under which they have been/are to be collected and provided for use. The purpose of this is to address and mitigate any potential legal and/or ethical implications of receipt and use of the materials as part of the research project, and to ensure that in doing so we align with best practice wherever possible.

The overarching areas of consideration are:

• Ethical review of provenance and sourcing of the material
• Legality of collection, transfer and use (national and international)

Each transfer of samples is undertaken according to a Research Collaboration Agreement or Material Transfer Agreement entered into by the Tree of Life collaborator, Genome Research Limited (operating as the Wellcome Sanger Institute) and in some circumstances other Tree of Life collaborators.

## Data Availability

European Nucleotide Archive: Millepora alcicornis. Accession number PRJEB83134; https://identifiers.org/ena.embl/PRJEB83134. The genome sequence is released openly for reuse. The *Millepora alcicornis* genome sequencing initiative is part of the Aquatic Symbiosis Genomics Project ( PRJEB43743) and the Sanger Institute Tree of Life Programme ( PRJEB43745). All raw sequence data and the assembly have been deposited in INSDC databases. Raw data and assembly accession identifiers are reported in Tables 1 and 2. Production code used in genome assembly at the WSI Tree of Life is available at https://github.com/sanger-tol . Table 6 lists software versions used in this study.
