# Supplementary material for: The chromosomal genome sequence of the Caribbean fire coral, Millepora alcicornis Linnaeus, 1758 (Anthoathecata: Milleporidae) and its associated microbial metagenome sequences
Source: Wellcome Open Res. 2026 Sep 17;11:685. [Version 1] doi: 10.12688/wellcomeopenres.27599.1 (PMC13740454; doi:10.12688/wellcomeopenres.27599.1)
Supplement: Peer review history

# Reviewer report: Reviewer response for version 1

**DOI:** 10.21956/wellcomeopenres.30381.r174792

**Published:** 2026-11-05

**Authors:** Jhonatas Sirino Monteiro

**Affiliations:** 1 Universidade de São Paulo, São Paulo, Brazil

This manuscript, “The chromosomal genome sequence of the Caribbean fire coral, *Millepora alcicornis* Linnaeus, 1758 (Anthoathecata: Milleporidae) and its associated microbial metagenome sequences,” presents the first reference genome for the hydrocoral *Millepora alcicornis*, a widely distributed Atlantic species with an important ecological role. The authors used state-of-the-art sequencing approaches (PacBio HiFi, Hi-C, and RNA-Seq) and genome-assembly software, enabling the generation of a high-quality reference genome for the species.

  

- **Is the rationale for creating the dataset(s) clearly described?**

Yes. The authors clearly describe the ecological relevance of the species as a reef-forming organism that provides habitat for a diversity of species and acts as a physical barrier by dissipating wave energy. Like scleractinian corals, the hydrocoral *Millepora alcicornis* maintains a symbiosis with zooxanthellae, which contributes to its autotrophic nutrition but makes the species susceptible to bleaching events, which have become more frequent over the last decade. Because of increasing concerns about its response to thermal stress, *M. alcicornis* has been the subject of transcriptomic and population-genetic studies, as the authors noted, making a reference genome of considerable interest to the scientific community. A reference genome would improve the use of transcriptomic data by enabling more accurate transcript assembly and would also facilitate population-genetic studies by providing a consolidated reference for more robust SNP calling, which provides the basis for analyses of connectivity and genetic diversity among *M. alcicornis* populations.

  

- **Are the protocols appropriate and is the work technically sound?**

Yes. The authors performed PacBio HiFi sequencing, which generates long, high-quality reads (approximately 10,679 bp per read). Combined with Hi-C sequencing, this approach makes it possible to produce a high-quality de novo chromosome-level genome assembly. The authors also applied a decontamination protocol to the assembly, given that the data may contain sequences derived from associated Symbiodiniaceae and prokaryotes. The genome was annotated using the Ensembl pipeline, which has previously been applied to model organisms such as human and mouse.

  

- **Are sufficient details of methods and materials provided to allow replication by others?**

Yes. The methodology is well described and documented.

  

- **Are the datasets clearly presented in a useable and accessible format?**

Partly. The authors have made the raw sequencing data available, as well as the FASTA file of the final *Millepora alcicornis* genome assembly and the annotation file. However, I was unable to find the files containing the MAGs. Given that the authors reported recovering four MAGs, I would expect the FASTA files of these genomes to be publicly available.

 Therefore, I consider the reference genome for *Millepora alcicornis* to be of substantial value to the scientific community. For this reason, I consider this Data Note suitable for indexing, considering the minor comments below.

  

1. BUSCO v6 now provides the cnidaria_odb12 lineage, which is considerably more specific (3.203 genes vs 954 genes from metazoa_odb10) and phylogenetically closer to *Millepora alcicornis* than metazoa_odb10. The odb12 datasets were released in 2025. I suggest that the authors update the BUSCO version used and reassess assembly completeness using the cnidaria_odb12 lineage.
2. I believe the manuscript should focus exclusively on describing the genome of *M. alcicornis*, as the metagenomic component is only briefly explored in the Data Note. The completeness of the metagenome-assembled genomes is very low, with the exception of one assembly that reached 93.37%. Moreover, unexpectedly, the most complete assembly could only be classified at the class level ( *Gammaproteobacteria*), whereas another assembly with much lower completeness could be classified to the genus level (Table 5). In addition, as noted above, the MAGs sequences are not publicly available. Therefore, I recommend that the associated metagenome not be presented in this Data Note.
3. As a suggestion, the authors could add Gene Ontology terms to the genome annotation and include a figure showing the most represented functional categories in the genome.
4. Could the authors provide the FastQC reports (or reports from a similar tool) as supplementary material, showing the quality metrics obtained for the sequencing runs?

Are sufficient details of methods and materials provided to allow replication by others?

Yes

Is the rationale for creating the dataset(s) clearly described?

Yes

Are the datasets clearly presented in a useable and accessible format?

Partly

Are the protocols appropriate and is the work technically sound?

Yes

Reviewer Expertise:

bioinformatics, genomics, transcriptomics, population genetics.

I confirm that I have read this submission and believe that I have an appropriate level of expertise to confirm that it is of an acceptable scientific standard.

# Reviewer report: Reviewer response for version 1

**DOI:** 10.21956/wellcomeopenres.30381.r174791

**Published:** 2026-10-24

**Authors:** Jean-Baptiste Ledoux

**Affiliations:** 1 CIIMAR/CIMAR, Centro Interdisciplinar de Investigação Marinha e Ambiental, Porto, Portugal

This data note, entitled « The chromosomal genome sequence of the Caribbean fire coral, Millepora alcicornis Linnaeus, 1758 (Anthoathecata: Milleporidae) and its associated microbial metagenome sequences” by Schizas and collaborators described the annotated chromosome level reference genome of *Millepora alcicornis*.

 The Authors used a hybrid strategy combining PacBioHifi, Hi-C and RNAsequencing to assemble a high-quality genome as supported by the related statistics.

 This genome is timely providing potentially valuable insights into the M. alcicornis molecular ecology and cnidarian evolution in general. I believe the manuscript is suitable for indexing considering the minor comments below.

 #1: *M. alcicornis* is a symbiotic species. Nevertheless, it is not clear how the Authors deal with this during the genome assembly. In addition, it would have been interesting to know the Symbiodinium species found in the samples used for the assembly.

 #2: How did the authors preserve the samples. Following the DNA extraction protocol, it seems that they used flash frozen tissue but this should be mentioned.

 #3: It would be interesting to know how many high quality annotated reference genomes are available in Hydrozoans, this will surely strengthened the interest of the paper.

 #4: “As coral reef degradation continues, calcium carbonate deposition by non-scleractinian organisms, such as Millepora, may become increasingly important for reef persistence, especially as the relative abundance of the reef-building taxa changes (Edmunds, 2022).” vs. “M. alcicornis is highly vulnerable to the same threats causing the decline of scleractinian corals. It is susceptible to bleaching events, where stressful conditions – primarily elevated sea surface temperatures – cause the expulsion of its symbiotic algae (Symbiodiniaceae).”

 The two sentences are contradictory to some point. I would stick on the second one and delete the first one.

 #5: How does the annotation fit with previously published de novo transcriptome assembly?

Are sufficient details of methods and materials provided to allow replication by others?

Yes

Is the rationale for creating the dataset(s) clearly described?

Yes

Are the datasets clearly presented in a useable and accessible format?

Yes

Are the protocols appropriate and is the work technically sound?

Yes

Reviewer Expertise:

biodiversity genomics, molecular and evolutionary genomics, habitat forming species.

I confirm that I have read this submission and believe that I have an appropriate level of expertise to confirm that it is of an acceptable scientific standard.
